# Crystal structures of binary compounds of meldonium 3-(1,1,1-tri­methyl­hydrazin-1-ium-2-yl)prop­ano­ate with sodium bromide and sodium iodide

**DOI:** 10.1107/S2056989018006977

**Published:** 2018-05-22

**Authors:** Alexander Y Nazarenko

**Affiliations:** a Chemistry Department, SUNY Buffalo State, 1300 Elmwood Ave, Buffalo, NY 14222, USA

**Keywords:** crystal structure, 3-(1,1,1-tri­methyl­ydrhazin-1-ium-2-yl)propano­ate, meldonium, sodium bromide, sodium iodide

## Abstract

3-(1,1,1-Tri­methyl­hydrazin-1-ium-2-yl)propano­ate (C_6_H_14_N_2_O_2_, **M**, more commonly known under its commercial names *Meldonium* or *Mildronate*) co-crystalizes with sodium bromide and sodium iodide forming polymeric hydrates poly[[tetra-μ-aqua-di­aqua­bis­[3-(1,1,1-tri­methyl­hydrazin-1-ium-2-yl)propano­ate]disodium] dibromide tetra­hydrate], [Na_2_(C_6_H_14_N_2_O_2_)_2_(H_2_O)_6_]Br_2_·4H_2_O, and poly[[di-μ-aqua-di­aqua­[μ-3-(1,1,1-tri­methyl­hydrazin-1-ium-2-yl)propano­ate]disodium] diiodide], [Na_2_(C_6_H_14_N_2_O_2_)_2_(H_2_O)_4_]I_2_. The coordination numbers of the sodium ions are 6; the coordination polyhedra can be described as distorted octa­hedra. Metal ions and **M** zwitterions are assembled into infinite layers *via* electrostatic inter­actions and hydrogen-bonded networks. These layers are connected *via* electrostatic attraction between halogenide ions and positive tri­methyl­hydrazinium groups into a three-dimensional structure.

## Abstract 2

3-(1,1,1-Tri­methyl­hydrazin-1-ium-2-yl)propano­ate (**M**, more commonly known under its commercial names *Meldonium* or *Mildronate*) co-crystalizes with sodium bromide and sodium iodide forming polymeric hydrates. Metal ions and **M** zwitterions are assembled into infinite layers *via* electrostatic inter­actions and hydrogen-bonded networks. These layers are connected *via* electrostatic attraction between halogenide ions and positive tri­methyl­hydrazinium groups into a three-dimensional structure.

## Chemical context   

3-(1,1,1-Tri­methyl­hydrazin-1-ium-2-yl)propano­ate (**M**), more commonly known under its commercial names such as *Meldonium* or *Mildronate*, was introduced by Grindeks (Latvia) as an anti-ischemic medication (Liepinsh *et al.*, 2017 ▸). The synthesis of **M** was originally described by Giller *et al.* (1975 ▸) and was improved in a number of patents and papers (Kalvins & Stonans, 2009 ▸; Kalvins *et al.*, 2014 ▸; Silva, 2013 ▸). Recently **M** achieved controversial publicity as a doping agent. As a result of its inclusion in the World Anti-Doping Agency List of Prohibited Substances, it attracted the attention of pharmaceutical and forensic chemists (Görgens *et al.*, 2015 ▸).

Binary compounds of **M** with various inorganic salts have been described in numerous **M**-related synthetic procedures (see above); their high stability was a challenge that was necessary to overcome for the preparation of pharmaceutically pure forms of **M**. The stability of a sodium iodide binary compound was given as an example in Silva (2013 ▸). The crystal structures of two such binary compounds, with sodium bromide (I) and with sodium iodide (II), are presented here.

## Structural commentary   

**Figure 1:**
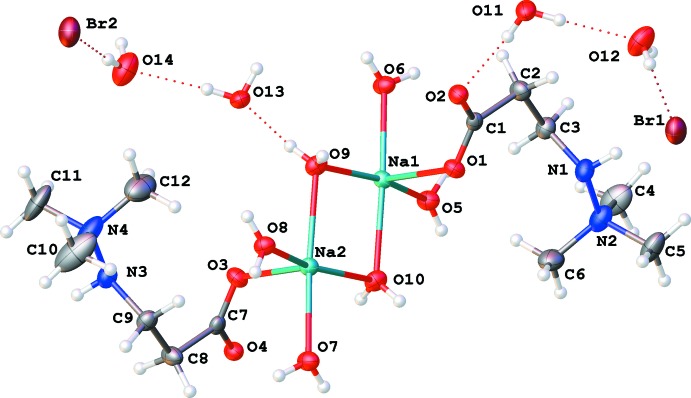
Labelling scheme of the asymmetric unit of compound (I) with 50% probability displacement ellipsoids.

**Figure 3:**
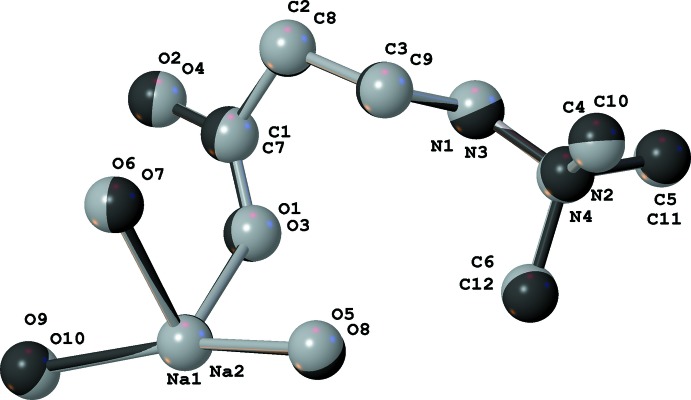
Overlay of the two organic fragments in (I) after inversion. The average deviation is 0.04 Å.

The labelling schemes for structures (I) and (II) are shown in Figs. 1 ▸ and 2 ▸. Mol­ecules of (I), which crystallize in an acentric space group, have a non-crystallographic inversion centre at 0.6238 (6) 0.744 (5) 0.5001 (2). This symmetry is visible in Fig. 1 ▸; it is also demonstrated by overlay of the two chemically equivalent moieties, after inversion of one of them (Fig. 3 ▸). Both Na ions have distorted octa­hedral environments (coordination number 6). The coordination sphere contains an anionic oxygen atom of a monodentate carb­oxy­lic group, two pairs of bridging O atoms of water mol­ecules (O5, O8, O9 and O10), and a terminal water mol­ecule (atoms O6 and O7 for Na1 and Na2 respectively). The shortest Na—O separations (Table 1 ▸) correspond to the anionic oxygens O1 and O3; the longest are opposite to the bridging atoms O5 and O8 (not shown in Fig. 1 ▸, but visible in Fig. 6 ▸).
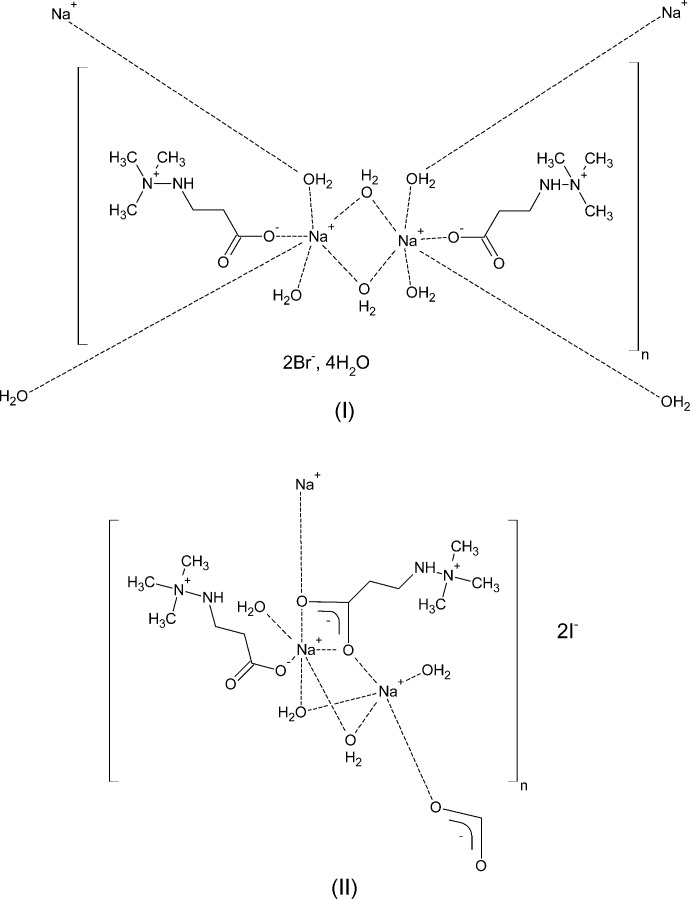


**Figure 4:**
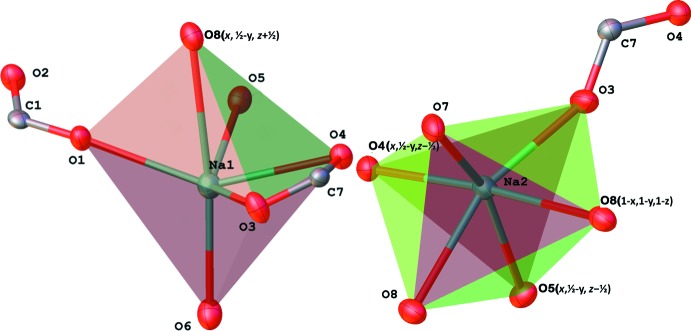
Coordination polyhedra of the sodium ions in (II).

The coordination polyhedra of the sodium ions in (II) are visibly different (Fig. 4 ▸, Table 2 ▸). Both have a distorted octa­hedral geometry and coordination number 6. The coordination polyhedron of Na1 contains an anionic oxygen atom O1 of a monodentate carb­oxy­lic group, atoms O3 and O4 of the bidentate carb­oxy­lic acid group, and three water mol­ecules O5, O6, and O8. The O8 atom, which forms three bridging contacts to three different sodium ions, shows a much longer separation from Na1 than any of the other coordinated oxygen atoms (Table 2 ▸).

The octa­hedral environment around Na2 in (II) (Fig. 4 ▸, Table 2 ▸) is less distorted: it consists of two bridging oxygen atoms O3 and O4 of two distinct carboxyl­ate groups and four water oxygen atoms. The shortest distance is Na2—O3 (involving carboxyl­ate group oxygens); the two longest again belong to the bridging O8 atoms (Table 2 ▸).

All zwitterions of **M** have approximately the same geometry (the two pseudo-inversion-symmetric zwitterions in the structure of (I) are nearly superimposable, Fig. 3 ▸). Both monodentate carboxyl­ates in (I) and that in (II) have slightly elongated C—O bonds for the oxygen atom bound to the corresponding Na ion (Tables 1 ▸ and 2 ▸). These bonds are slightly longer than the corresponding bonds in **M** monohydrate and dihydrate [1.258 (2) and 1.2618 (9) Å, respectively; CCDC entries CCDC 1822460 and 1822463; Naza­renko, 2018 ▸). This relatively small change could be inter­preted as a shift of of the anionic charge towards the sodium-bound oxygen atom. The carbon–oxygen bond lengths within the bidenate carboxyl­ate groups in (II) are essentially identical within two standard deviations.

All N—N bond distances are around 1.47 Å (Tables 1 ▸ and 2 ▸) and are within experimental error indistinguishable from the average value [1.468 (2) Å] for known low-temperature single-crystal structures of **M** (CCDC 1822460–1822463; Naza­renko, 2018 ▸), but significantly shorter than the value reported for room temperature (1.49 Å; Kemme *et al.*, 1983 ▸).

**Figure 5:**
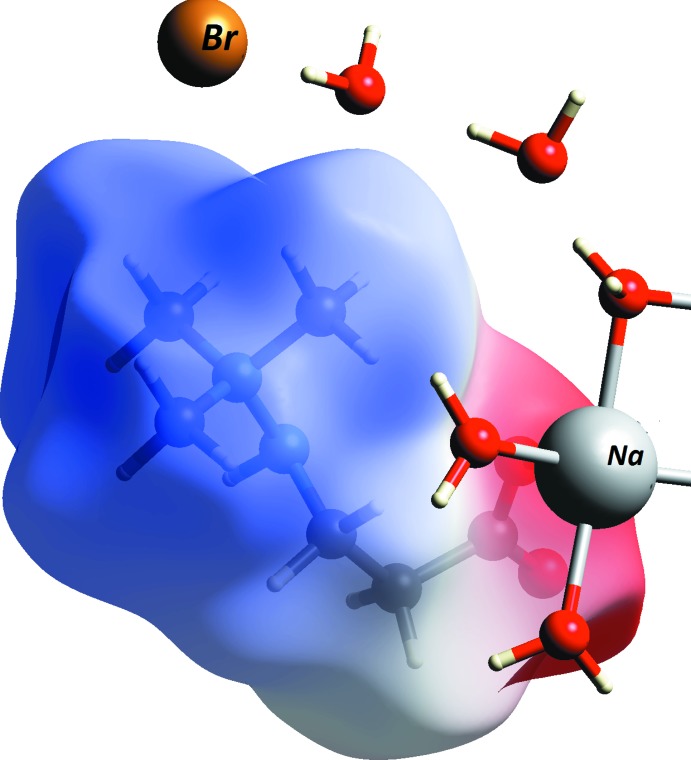
Hirshfeld surface of the zwitterion with electrostatic potential plotted using CrystalExplorer*17* (Turner *et al.*, 2017 ▸). Red – negative, blue – positive.

The distribution of the Hirshfeld surface electrostatic potential of the zwitterion (Fig. 5 ▸) shows that only a small area around the carboxyl oxygen atoms is negatively charged: the remaining Hirshfeld surface has positive electrostatic potential. This makes this area attractive for anions, with the N—H group of the hydrazine fragment available as a donor of an electrostatically enhanced hydrogen bond. The lone-pair density of the same hydrazine nitro­gen atom is not sufficient to overcome the total positive charge of the tri­methyl­hydrazinium fragment and does not act as a hydrogen-bond acceptor.

## Supra­molecular features   

**Figure 6:**
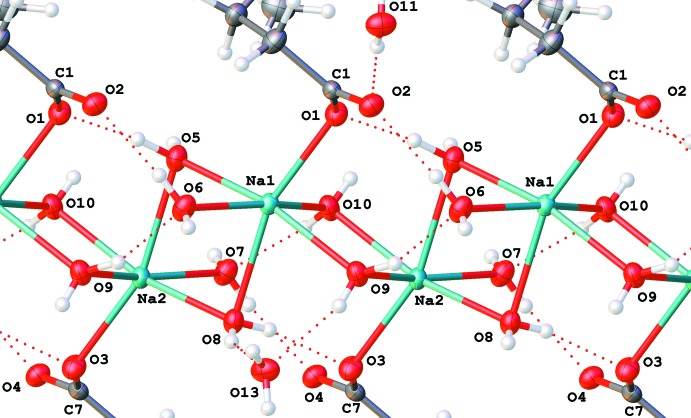
The infinite chain of hydrated sodium ions along the [010] axis in (I).

In the structure of (I), the coordination polyhedra of the sodium ions are connected by common edges (a pair of bridging water mol­ecules, O5 and O8, and O9 and O10), forming an infinite chain of ions along the [010] vector (Fig. 6 ▸). In addition to Na⋯O inter­actions, this chain is supported by six hydrogen bonds (Table 3 ▸): O6—H6*B*⋯O2, O5—H5*A*⋯O1, O8—H8*B*⋯O3, O7—H7*B*⋯O4, O9—H9*A*⋯O6 and O10—H10*B*⋯O7. The first four of them, connecting the anionic oxygen atoms of the carb­oxy­lic groups, are electrostatically enhanced.

**Figure 7:**
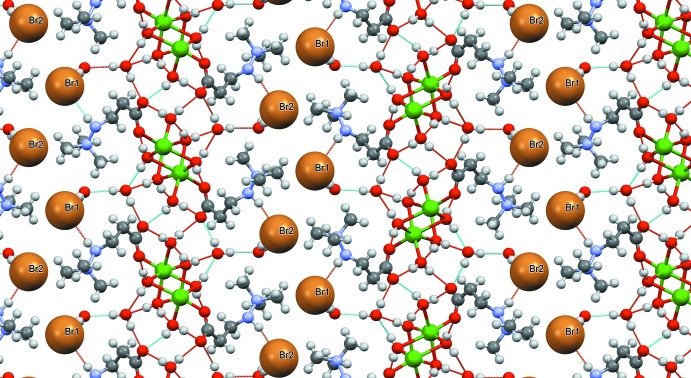
Packing of (I). View along the [010] axis. Sodium ions are green.

Each bromide ion forms a hydrogen bond with a hydrazine N—H group. In addition, each of them forms two hydrogen bonds with neighboring water mol­ecules (O12 and O14), thus forming two more infinite chains in the [010] direction. Water mol­ecules O11 and O13 form bridges between the cation chain and the ‘bromide’ chains as hydrogen-bond donors; they are also acceptors of four hydrogen bonds from the water mol­ecules O5 and O10, and O8 and O9 respectively. These hydrogen bonds connect chains into a two-dimensional network. Two more enhanced hydrogen bonds (Table 3 ▸), O7—H7*A*⋯O2 and O6—H6*A*⋯O4, also connect neighboring chains. The resulting network forms a layer in the (001) plane with the bromide ions and tri­methyl­ammonium groups forming each side (Fig. 7 ▸). These layers are bound together *via* electrostatic inter­action of the corresponding positive and negative ions; no short intra­layer contacts are visible.

**Figure 8:**
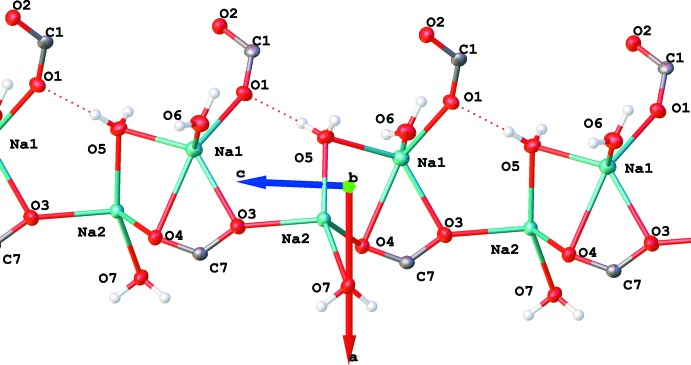
The infinite chain of hydrated sodium ions along the [001] axis in (II).

**Figure 9:**
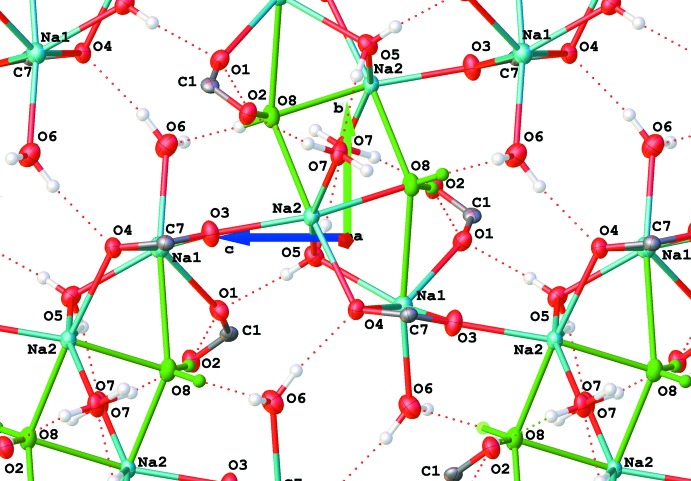
Chains in the structure of (II) are connected *via* atom O8 (in green) and a network of hydrogen bonds (dashed lines).

**Figure 10:**
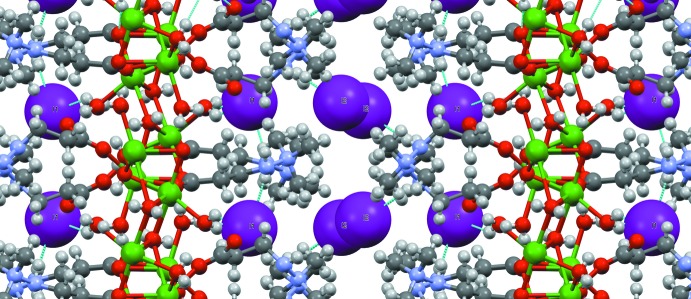
Packing of (II). View along the [001] axis. Sodium ions are green.

In the structure of (II), the coordination polyhedra of the sodium ions are bridged *via* the bidentate carboxyl­ate group to form an infinite chain along the [001] axis (Fig. 8 ▸). The water mol­ecule O5 provides an additional bridge, stabilizing the chain. These chains are inter­connected in the (100) plane with the help of weaker (and longer by almost 0.5 Å) Na⋯O8 contacts (Fig. 9 ▸). An array of hydrogen bonds (Table 4 ▸, Fig. 9 ▸) additionally stabilizes the resulting layer. As in compound (I), both iodide ions are connected to zwitterions **M** *via* N—H⋯I^−^ hydrogen bonds. In addition, ion I1 is an acceptor of two hydrogen bonds with water mol­ecules (O6—H6*A*⋯I1 and O7—H7*A*⋯I1, see Table 4 ▸). In absence of neighboring water mol­ecules, two CH groups of the tri­methyl­ammonium fragment form close contacts with the ion I2. As in structure (I), the layers are tied together by the electrostatic inter­action of the corresponding positive and negative ions; no short intra­layer contacts are visible (Fig. 10 ▸).

## Database survey   

Prior to 2018, the only meldonium-related single-crystal structure in the Cambridge Structural Database (Groom *et al.*, 2016 ▸, CSD Version 5.39) had been a crystal structure of the dihydrate form (refcode CABVOQ; Kemme *et al.*, 1983 ▸)) measured at room temperature with no experimental positions for hydrogen atoms. Hydrates of **M** also were also studied using powder X-ray diffraction (Zvirgzdiņš *et al.*, 2011 ▸; Bērziņš & Actiņš, 2014 ▸). Meldonium is closely related to betaines, a wide class of zwitterionic compounds with an onium atom that bears no hydrogen atoms and that is not adjacent to the anionic atom. The parent compound of the betaine class, *N*,*N*,*N*-tri­methyl­glycine (TMG), has a very rich crystal chemistry: the CSD (Version 5.39) contains 217 different structures of its compounds. There are several known crystal structures of TMG binary compounds with potassium iodide (HIPQIG; Andrade *et al.*, 1999 ▸), rubidium iodide (NEMKIZ; Andrade *et al.*, 2001 ▸), potassium bromide (WIQPUH01; Andrade *et al.*, 2000 ▸) and sodium bromide (JAZNEE; Rodrigues *et al.*, 2005 ▸). These compounds show features similar to those of their meldonium analogs: infinite chains of hydrated alkali metal cations and layers of tri­methyl­ammonium groups. The obvious differences are the absence of N—H⋯*X* ^−^ hydrogen bonds and the much smaller size of the organic domain.

## Synthesis and crystallization   

Preparation and properties of binary compounds of **M** with sodium halogenides are described in detail in Giller *et al.* (1975 ▸) and Silva (2013 ▸). Commercial **M** dihydrate was received from Grindeks (Latvia) and recrystallized from propanol-2. Equimolar amounts of it were mixed with sodium iodide and sodium bromide in aqueous ethanol; subsequent slow evaporation yielded crystals suitable for single–crystal X-ray experiments. IR spectra (FTIR–ATR, cm^−1^) are very similar to those of **M** dihydrate. (I): 3399 (H_2_O), 1571, 1483, 1402, 1320; (II): 3350, 3180 (H_2_O), 1568, 1480, 1405, 1317, 1088, 816; **M** dihydrate: 3201 (H_2_O), 1577, 1484, 1404, 1320, 1090, 816.

## Refinement   

Crystal data, data collection and structure refinement details are summarized in Table 5 ▸.

Structure (I) was was solved and refined in an achiral space group; the large Flack parameter prompted twin refinement as a two-component inversion twin [0.75 (1):0.25 (1)] with twin matrix [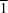 0 0, 0 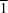 0, 0 0 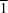]. Reflections in (II) were processed as a two-domain [0.668 (1):0.332 (1) ratio] non-merohedral twin with twin matrix [1.000 0.000 0.000, 0.000 −1.000 0.000, −0.146 0.000 −1.000]; domain 2 is rotated from the first domain by 180.0° about the reciprocal axis 1.000 −0.001 −0.073 or the real axis 1.000 0.000 0.002 (*CELL_NOW*; Sheldrick, 2008 ▸).

In the structure of (I) distances O6—H6*A*, O6—H6*B*, O7—H7*A*, O7—H7*B*, O8—H8*A*, and O8—H8*B*; O11—H11*D*, O11—H11*E*, O12—H12*E*, O12—H12*D*, O13—H13*A*, and O13—H13*B*; O14—H14*A* and O14—H14*B* were restrained to be equal with an effective standard deviation of 0.02 Å. Distances N1—H1 and N3—H3 were also restrained to be equal with an effective standard deviation of 0.02 Å; *U*_iso_(H) = 1.5*U*_iso_(N).

In the structure of (II), water mol­ecules O6 and O7 were refined as rotating groups (AFIX 7). The positions and isotropic displacement parameters of the hydrazinium hydrogen atoms were refined.

In both structures, methyl­ene hydrogen atoms were refined with riding coordinates and with *U*_iso_(H) = 1.2 *U*_iso_(C); methyl hydrogen atoms were refined as rotating idealized methyl groups and with *U*_iso_(H) = 1.5*U*_iso_(C). Hydrogen atoms of water mol­ecules were refined in an isotropic approximation with *U*_iso_(H) = 1.5*U*_iso_(O).

## Supplementary Material

Crystal structure: contains datablock(s) I, II. DOI: 10.1107/S2056989018006977/zl2729sup1.cif

Structure factors: contains datablock(s) I. DOI: 10.1107/S2056989018006977/zl2729Isup2.hkl

Supporting information file. DOI: 10.1107/S2056989018006977/zl2729Isup4.cdx

Structure factors: contains datablock(s) II. DOI: 10.1107/S2056989018006977/zl2729IIsup3.hkl

Supporting information file. DOI: 10.1107/S2056989018006977/zl2729IIsup5.cdx

CCDC references: 1841872, 1841871

Additional supporting information:  crystallographic information; 3D view; checkCIF report

## Figures and Tables

**Figure 2:**
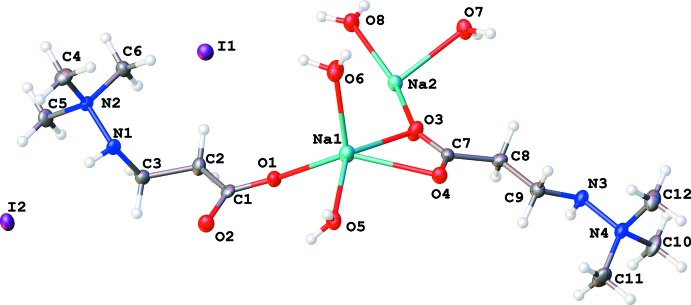
Labelling scheme of the asymmetric unit of compound (II) with 50% probability displacement ellipsoids.

**Table 1:** Selected bond lengths (Å) for (I)

Na1—O1	2.367 (4)	Na2—O8	2.364 (3)
Na1—O5	2.368 (3)	Na2—O9	2.361 (3)
Na1—O6	2.369 (3)	Na2—O10	2.449 (4)
Na1—O8^i^	2.517 (4)	O1—C1	1.241 (5)
Na1—O9	2.442 (4)	O2—C1	1.281 (5)
Na1—O10	2.361 (4)	O3—C7	1.249 (5)
Na2—O3	2.359 (4)	O4—C7	1.282 (5)
Na2—O5^ii^	2.543 (4)	N1—N2	1.471 (6)
Na2—O7	2.368 (3)	N3—N4	1.466 (6)

Symmetry codes: (i) 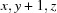; (ii) 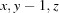.

**Table 2:** Selected bond lengths (Å) for (II)

Na1—O1	2.462 (3)	Na2—O7	2.372 (3)
Na1—O3	2.374 (3)	Na2—O8^iii^	2.569 (4)
Na1—O4	2.552 (3)	Na2—O8	2.510 (3)
Na1—O5	2.351 (3)	O1—C1	1.274 (4)
Na1—O6	2.385 (4)	O2—C1	1.247 (4)
Na1—O8^i^	2.857 (4)	O3—C7	1.256 (5)
Na2—O3	2.315 (3)	O4—C7	1.264 (5)
Na2—O4^ii^	2.431 (3)	N1—N2	1.478 (4)
Na2—O5^ii^	2.373 (3)	N3—N4	1.476 (4)

Symmetry codes: (i) 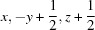; (ii) 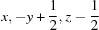; (iii) 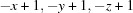.

**Table 3:** Hydrogen-bond geometry (Å, °) for (I)

*D*—H⋯*A*	*D*—H	H⋯*A*	*D*⋯*A*	*D*—H⋯*A*
O5—H5*A*⋯O1^i^	0.97 (6)	1.79 (6)	2.746 (4)	172 (6)
O5—H5*B*⋯O11^iii^	0.82 (6)	2.02 (6)	2.819 (5)	167 (6)
O6—H6*A*⋯O4^iv^	0.80 (3)	2.06 (3)	2.845 (5)	165 (6)
O6—H6*B*⋯O2^i^	0.82 (3)	1.91 (3)	2.732 (4)	175 (6)
O7—H7*A*⋯O2^v^	0.82 (3)	2.05 (3)	2.856 (5)	165 (6)
O7—H7*B*⋯O4^ii^	0.80 (3)	1.94 (3)	2.731 (4)	169 (6)
O8—H8*A*⋯O13^ii^	0.81 (3)	2.06 (3)	2.815 (5)	155 (5)
O8—H8*B*⋯O3^ii^	0.81 (3)	1.95 (3)	2.754 (4)	168 (7)
O9—H9*A*⋯O6^ii^	0.93 (6)	1.96 (6)	2.852 (4)	160 (5)
O9—H9*B*⋯O13	0.79 (6)	2.01 (6)	2.772 (5)	160 (6)
O10—H10*A*⋯O11^v^	0.78 (6)	2.00 (6)	2.771 (5)	167 (6)
O10—H10*B*⋯O7^i^	0.90 (6)	1.99 (6)	2.853 (4)	158 (5)
O11—H11*D*⋯O12	0.80 (3)	1.94 (3)	2.744 (6)	179 (7)
O11—H11*E*⋯O2	0.80 (3)	1.92 (3)	2.719 (5)	174 (9)
O13—H13*A*⋯O14	0.80 (3)	1.95 (3)	2.733 (6)	170 (6)
O13—H13*B*⋯O4^iv^	0.80 (3)	1.94 (3)	2.727 (5)	168 (9)
N1—H1⋯Br1^i^	0.83 (5)	2.57 (5)	3.379 (5)	167 (5)
N3—H3⋯Br2^v^	0.84 (5)	2.57 (5)	3.394 (5)	169 (5)
O12—H12*D*⋯Br1^i^	0.80 (5)	2.52 (6)	3.316 (4)	172 (6)
O12—H12*E*⋯Br1	0.80 (5)	2.49 (6)	3.289 (4)	177 (8)
O14—H14*A*⋯Br2^i^	0.87 (7)	2.47 (7)	3.323 (5)	168 (7)
O14—H14*B*⋯Br2	0.87 (6)	2.41 (6)	3.281 (5)	175 (6)

Symmetry codes: (i) 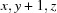; (ii) 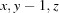; (iii) 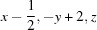; (iv) 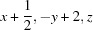; (v) 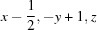.

**Table 4:** Hydrogen-bond geometry (Å, °) for (II)

*D*—H⋯*A*	*D*—H	H⋯*A*	*D*⋯*A*	*D*—H⋯*A*
O5—H5*B*⋯O1^i^	0.85 (6)	1.89 (6)	2.741 (4)	175 (6)
O7—H7*B*⋯O2^iv^	0.91	1.73	2.629 (4)	169
O8—H8*A*⋯O1^ii^	0.85 (6)	2.05 (6)	2.815 (4)	149 (6)
N1—H1⋯I2	0.82 (6)	2.87 (6)	3.688 (4)	177 (5)
N3—H3⋯I1^v^	0.92 (6)	2.76 (6)	3.650 (3)	161 (5)
O5—H5*A*⋯O7^v^	0.86 (6)	2.00 (6)	2.846 (4)	172 (4)
O6—H6*A*⋯I1	0.89	2.64	3.518 (3)	166
O6—H6*B*⋯O4^vi^	0.89	1.95	2.825 (4)	168
O7—H7*A*⋯I1^iii^	0.91	2.78	3.548 (3)	143
O8—H8*B*⋯O6^iii^	0.86 (7)	2.13 (7)	2.989 (5)	175 (5)
C3—H3*A*⋯I1^ii^	0.99	3.01	3.920 (4)	154
C11—H11*B*⋯I2^vii^	0.98	3.02	3.975 (4)	165
C12—H12*C*⋯I1^vi^	0.98	2.99	3.952 (5)	167

Symmetry codes: (i) 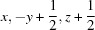; (ii) 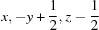; (iii) 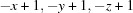; (iv) 
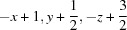
; (v) 
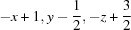
; (vi) 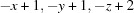; (vii) 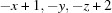.

**Table 5:** Experimental details

	(I)	(II)
Crystal data		
Chemical formula	[Na_2_(C_6_H_14_N_2_O_2_)_2_(H_2_O)_6_]Br_2_·4H_2_O	[Na_2_(C_6_H_14_N_2_O_2_)_2_(H_2_O)_4_]·I_2_
*M*_r_	678.34	664.23
Crystal system, space group	Orthorhombic, *P* *c* *a*2_1_	Monoclinic, *P*2_1_/*c*
Temperature (K)	173	173
*a*, *b*, *c* (Å)	16.5181 (8), 5.5262 (3), 33.2605 (16)	19.7455 (11), 11.4530 (7), 10.9733 (7)
α, β, γ (°)	90, 90, 90	90, 92.382 (2), 90
*V* (Å^3^)	3036.1 (3)	2479.4 (3)
*Z*	4	4
Radiation type	Mo *K*α	Mo *K*α
μ (mm^−1^)	2.76	2.61
Crystal size (mm)	0.65 × 0.13 × 0.09	0.3 × 0.2 × 0.07

Data collection		
Diffractometer	Bruker PHOTON-100 CMOS	Bruker PHOTON-100 CMOS
Absorption correction	Numerical (*SADABS*; Krause *et al.*, 2015 ▸)	Multi-scan (*TWINABS*; Krause *et al.*, 2015 ▸)
*T*_min_, *T*_max_	0.217, 0.635	0.301, 0.431
No. of measured, independent and observed [*I* > 2σ(*I*)] reflections	117729, 6969, 6121	5475, 5475, 5012
*R*_int_	0.044	0.048
(sin θ/λ)_max_ (Å^−1^)	0.650	0.641

Refinement		
*R*[*F* ^2^ > 2σ(*F* ^2^)], *wR*(*F* ^2^), *S*	0.032, 0.078, 1.03	0.026, 0.057, 1.17
No. of reflections	6969	5475
No. of parameters	380	286
No. of restraints	33	0
H-atom treatment	H atoms treated by a mixture of independent and constrained refinement	H atoms treated by a mixture of independent and constrained refinement
Δρ_max_, Δρ_min_ (e Å^−3^)	0.71, −0.38	0.70, −0.55
Absolute structure	Refined as an inversion twin	–
Absolute structure parameter	0.250 (10)	–

Computer programs: *APEX2* and *SAINT* (Bruker, 2016 ▸), *SHELXT* (Sheldrick, 2015*a* ▸), *SHELXL2016* (Sheldrick, 2015*b* ▸), *OLEX2* (Dolomanov *et al.*, 2009 ▸) and *CrystalExplorer17* (Turner *et al.*, 2017 ▸).

## supplementary crystallographic information

### Poly[[tetra-µ-aqua-diaquabis[3-(1,1,1-trimethylhydrazin-1-ium-2-yl)\ propanoate]disodium] dibromide tetrahydrate] (I) Crystal data

**Table:**

[Na_2_(C_6_H_14_N_2_O_2_)_2_(H_2_O)_6_]Br_2_·4H_2_O	*D*_x_ = 1.484 Mg m^−^^3^
*M**_r_* = 678.34	Mo *K*α radiation, λ = 0.71073 Å
Orthorhombic, *P**c**a*2_1_	Cell parameters from 9077 reflections
*a* = 16.5181 (8) Å	θ = 3.1–27.4°
*b* = 5.5262 (3) Å	µ = 2.76 mm^−^^1^
*c* = 33.2605 (16) Å	*T* = 173 K
*V* = 3036.1 (3) Å^3^	Needle, colourless
*Z* = 4	0.65 × 0.13 × 0.09 mm
*F*(000) = 1408	

### Poly[[tetra-µ-aqua-diaquabis[3-(1,1,1-trimethylhydrazin-1-ium-2-yl)\ propanoate]disodium] dibromide tetrahydrate] (I) Data collection

**Table:**

Bruker PHOTON-100 CMOS diffractometer	6121 reflections with *I* > 2σ(*I*)
Radiation source: sealedtube	*R*_int_ = 0.044
φ and ω scans	θ_max_ = 27.5°, θ_min_ = 3.1°
Absorption correction: numerical (SADABS; Krause *et al.*, 2015)	*h* = −21→21
*T*_min_ = 0.217, *T*_max_ = 0.635	*k* = −7→7
117729 measured reflections	*l* = −43→43
6969 independent reflections	

### Poly[[tetra-µ-aqua-diaquabis[3-(1,1,1-trimethylhydrazin-1-ium-2-yl)\ propanoate]disodium] dibromide tetrahydrate] (I) Refinement

**Table:**

Refinement on *F*^2^	Hydrogen site location: mixed
Least-squares matrix: full	H atoms treated by a mixture of independent and constrained refinement
*R*[*F*^2^ > 2σ(*F*^2^)] = 0.032	*w* = 1/[σ^2^(*F*_o_^2^) + (0.0417*P*)^2^ + 2.0085*P*] where *P* = (*F*_o_^2^ + 2*F*_c_^2^)/3
*wR*(*F*^2^) = 0.078	(Δ/σ)_max_ = 0.001
*S* = 1.02	Δρ_max_ = 0.71 e Å^−^^3^
6969 reflections	Δρ_min_ = −0.38 e Å^−^^3^
380 parameters	Absolute structure: Refined as an inversion twin
33 restraints	Absolute structure parameter: 0.250 (10)
Primary atom site location: dual	

### Poly[[tetra-µ-aqua-diaquabis[3-(1,1,1-trimethylhydrazin-1-ium-2-yl)\ propanoate]disodium] dibromide tetrahydrate] (I) Special details

**Table:**

Geometry. All esds (except the esd in the dihedral angle between two l.s. planes) are estimated using the full covariance matrix. The cell esds are taken into account individually in the estimation of esds in distances, angles and torsion angles; correlations between esds in cell parameters are only used when they are defined by crystal symmetry. An approximate (isotropic) treatment of cell esds is used for estimating esds involving l.s. planes.
Refinement. Refined as a 2-component inversion twin

### Poly[[tetra-µ-aqua-diaquabis[3-(1,1,1-trimethylhydrazin-1-ium-2-yl)\ propanoate]disodium] dibromide tetrahydrate] (I) Fractional atomic coordinates and isotropic or equivalent isotropic displacement parameters (Å^2^)

**Table:**

	*x*	*y*	*z*	*U*_iso_*/*U*_eq_	
Br1	0.92047 (3)	−0.03168 (8)	0.28604 (2)	0.03549 (13)	
Br2	0.82608 (3)	0.44931 (9)	0.71406 (2)	0.04007 (14)	
Na1	0.67706 (11)	0.9937 (3)	0.48342 (7)	0.0182 (5)	
Na2	0.57098 (11)	0.4989 (3)	0.51722 (7)	0.0186 (5)	
O1	0.73577 (16)	0.7342 (5)	0.43488 (9)	0.0229 (6)	
O2	0.8552 (2)	0.5504 (5)	0.44213 (11)	0.0240 (7)	
O3	0.51258 (17)	0.7566 (5)	0.56578 (9)	0.0243 (6)	
O4	0.39361 (19)	0.9443 (5)	0.55827 (11)	0.0226 (6)	
O5	0.63384 (19)	1.3548 (5)	0.45166 (9)	0.0237 (6)	
H5A	0.671 (4)	1.481 (10)	0.444 (2)	0.036*	
H5B	0.594 (3)	1.388 (11)	0.4385 (17)	0.036*	
O6	0.78937 (18)	1.2455 (5)	0.49791 (10)	0.0249 (6)	
H6A	0.825 (2)	1.210 (10)	0.5134 (14)	0.037*	
H6B	0.809 (3)	1.330 (9)	0.4802 (14)	0.037*	
O7	0.45879 (18)	0.2480 (5)	0.50216 (10)	0.0255 (6)	
H7A	0.423 (3)	0.308 (9)	0.4882 (15)	0.038*	
H7B	0.435 (3)	0.173 (10)	0.5193 (14)	0.038*	
O8	0.61447 (17)	0.1366 (5)	0.54820 (9)	0.0222 (6)	
H8A	0.651 (2)	0.124 (10)	0.5642 (13)	0.033*	
H8B	0.584 (3)	0.022 (7)	0.550 (2)	0.033*	
O9	0.70252 (18)	0.6523 (5)	0.52830 (10)	0.0240 (6)	
H9A	0.729 (3)	0.506 (10)	0.5247 (19)	0.036*	
H9B	0.717 (3)	0.707 (10)	0.5492 (19)	0.036*	
O10	0.54562 (19)	0.8403 (5)	0.47200 (10)	0.0241 (6)	
H10A	0.530 (3)	0.776 (10)	0.4526 (18)	0.036*	
H10B	0.507 (4)	0.945 (10)	0.480 (2)	0.036*	
N1	0.7581 (3)	0.9463 (7)	0.34771 (14)	0.0243 (9)	
H1	0.792 (3)	0.949 (10)	0.3293 (16)	0.037*	
N2	0.6865 (3)	1.0657 (6)	0.33042 (12)	0.0308 (9)	
N3	0.4899 (3)	0.5316 (7)	0.65254 (15)	0.0254 (9)	
H3	0.454 (3)	0.544 (10)	0.6701 (16)	0.038*	
N4	0.5581 (3)	0.3991 (7)	0.67014 (13)	0.0398 (10)	
C1	0.8094 (2)	0.7177 (7)	0.42815 (12)	0.0180 (8)	
C2	0.8516 (3)	0.9122 (8)	0.40294 (14)	0.0266 (9)	
H2A	0.886964	1.009290	0.420772	0.032*	
H2B	0.886701	0.831769	0.382845	0.032*	
C3	0.7943 (3)	1.0812 (7)	0.38121 (13)	0.0240 (9)	
H3A	0.751492	1.138148	0.399790	0.029*	
H3B	0.824043	1.223813	0.370938	0.029*	
C4	0.6963 (4)	1.3329 (8)	0.32444 (18)	0.0457 (14)	
H4A	0.697253	1.413826	0.350657	0.069*	
H4B	0.650888	1.394886	0.308527	0.069*	
H4C	0.747182	1.364707	0.310239	0.069*	
C5	0.6719 (4)	0.9498 (8)	0.2904 (2)	0.0416 (14)	
H5C	0.719071	0.975629	0.273010	0.062*	
H5D	0.623950	1.022298	0.277842	0.062*	
H5E	0.663080	0.775844	0.293987	0.062*	
C6	0.6167 (4)	1.0165 (10)	0.3575 (2)	0.0382 (14)	
H6C	0.607611	0.841579	0.359257	0.057*	
H6D	0.568050	1.095072	0.346802	0.057*	
H6E	0.628344	1.080630	0.384387	0.057*	
C7	0.4383 (2)	0.7743 (7)	0.57220 (12)	0.0190 (8)	
C8	0.3960 (3)	0.5814 (8)	0.59707 (14)	0.0270 (9)	
H8C	0.361903	0.662841	0.617476	0.032*	
H8D	0.359465	0.488736	0.579189	0.032*	
C9	0.4512 (3)	0.4059 (7)	0.61824 (13)	0.0244 (9)	
H9C	0.419698	0.265265	0.628036	0.029*	
H9D	0.493099	0.346329	0.599409	0.029*	
C10	0.5447 (5)	0.1391 (10)	0.6751 (2)	0.073 (2)	
H10C	0.495470	0.112758	0.691030	0.110*	
H10D	0.591059	0.067082	0.689066	0.110*	
H10E	0.538397	0.063338	0.648684	0.110*	
C11	0.5722 (5)	0.5108 (9)	0.7105 (2)	0.052 (2)	
H11A	0.584574	0.683111	0.707269	0.078*	
H11B	0.617855	0.429848	0.723737	0.078*	
H11C	0.523504	0.492499	0.727074	0.078*	
C12	0.6308 (5)	0.4501 (13)	0.6429 (3)	0.0527 (18)	
H12A	0.621070	0.379778	0.616269	0.079*	
H12B	0.679559	0.377958	0.654625	0.079*	
H12C	0.638291	0.625353	0.640292	0.079*	
O11	1.0017 (2)	0.4552 (7)	0.40834 (13)	0.0284 (8)	
H11D	0.999 (4)	0.458 (11)	0.3843 (8)	0.043*	
H11E	0.957 (3)	0.488 (11)	0.417 (3)	0.043*	
O12	0.9948 (3)	0.4654 (7)	0.32594 (15)	0.0441 (10)	
H12D	0.973 (4)	0.586 (9)	0.318 (2)	0.066*	
H12E	0.975 (4)	0.348 (9)	0.316 (2)	0.066*	
O13	0.7469 (2)	0.9506 (6)	0.59158 (13)	0.0272 (8)	
H13A	0.752 (4)	0.933 (11)	0.6152 (8)	0.041*	
H13B	0.793 (2)	0.976 (11)	0.585 (3)	0.041*	
O14	0.7528 (3)	0.9447 (8)	0.67369 (14)	0.0489 (11)	
H14A	0.777 (5)	1.077 (10)	0.681 (3)	0.073*	
H14B	0.771 (4)	0.817 (10)	0.686 (2)	0.073*	

### Poly[[tetra-µ-aqua-diaquabis[3-(1,1,1-trimethylhydrazin-1-ium-2-yl)\ propanoate]disodium] dibromide tetrahydrate] (I) Atomic displacement parameters (Å^2^)

**Table:**

	*U*^11^	*U*^22^	*U*^33^	*U*^12^	*U*^13^	*U*^23^
Br1	0.0441 (3)	0.0355 (2)	0.0268 (3)	−0.0014 (2)	0.0046 (2)	−0.0016 (3)
Br2	0.0516 (3)	0.0410 (2)	0.0277 (3)	0.0037 (2)	0.0026 (3)	0.0051 (3)
Na1	0.0180 (10)	0.0155 (7)	0.0211 (14)	0.0004 (5)	0.0014 (8)	0.0006 (6)
Na2	0.0197 (11)	0.0147 (7)	0.0213 (14)	−0.0003 (6)	−0.0004 (8)	0.0001 (5)
O1	0.0191 (14)	0.0220 (14)	0.0276 (15)	−0.0005 (11)	0.0029 (12)	0.0007 (11)
O2	0.0220 (16)	0.0263 (14)	0.0235 (18)	0.0009 (13)	−0.0005 (13)	0.0040 (13)
O3	0.0198 (15)	0.0244 (14)	0.0287 (16)	0.0009 (12)	0.0051 (11)	0.0023 (12)
O4	0.0200 (16)	0.0248 (14)	0.0230 (17)	0.0007 (12)	−0.0010 (13)	0.0050 (13)
O5	0.0232 (15)	0.0182 (13)	0.0297 (16)	0.0008 (12)	−0.0024 (13)	0.0033 (12)
O6	0.0224 (16)	0.0239 (14)	0.0285 (17)	−0.0023 (12)	−0.0057 (12)	0.0067 (12)
O7	0.0233 (16)	0.0245 (15)	0.0288 (16)	−0.0024 (12)	−0.0030 (12)	0.0071 (12)
O8	0.0227 (15)	0.0179 (13)	0.0261 (16)	−0.0018 (11)	−0.0028 (12)	0.0017 (12)
O9	0.0267 (15)	0.0192 (14)	0.0262 (16)	0.0017 (12)	−0.0042 (13)	−0.0026 (12)
O10	0.0273 (16)	0.0183 (14)	0.0267 (16)	0.0013 (12)	−0.0041 (12)	−0.0031 (11)
N1	0.030 (2)	0.0216 (17)	0.021 (2)	0.0059 (16)	−0.0010 (17)	0.0027 (15)
N2	0.046 (2)	0.0195 (17)	0.027 (2)	0.0025 (16)	−0.0092 (17)	0.0028 (15)
N3	0.031 (2)	0.0231 (17)	0.022 (2)	0.0029 (16)	−0.0017 (18)	−0.0007 (15)
N4	0.061 (3)	0.028 (2)	0.031 (2)	0.012 (2)	−0.021 (2)	−0.0027 (17)
C1	0.022 (2)	0.0159 (17)	0.0157 (19)	−0.0030 (14)	−0.0003 (15)	−0.0020 (14)
C2	0.025 (2)	0.027 (2)	0.028 (2)	−0.0044 (18)	−0.0001 (19)	0.0064 (18)
C3	0.029 (2)	0.0203 (19)	0.023 (2)	0.0013 (17)	−0.0015 (17)	0.0030 (17)
C4	0.066 (4)	0.018 (2)	0.053 (3)	0.002 (2)	−0.019 (3)	0.009 (2)
C5	0.066 (4)	0.032 (2)	0.027 (3)	−0.004 (2)	−0.016 (3)	−0.001 (2)
C6	0.026 (3)	0.049 (3)	0.039 (4)	0.004 (2)	−0.004 (3)	−0.002 (2)
C7	0.024 (2)	0.0187 (18)	0.0142 (19)	−0.0032 (15)	−0.0014 (15)	−0.0030 (14)
C8	0.021 (2)	0.030 (2)	0.030 (2)	−0.0014 (18)	0.0002 (18)	0.0111 (19)
C9	0.028 (2)	0.0211 (19)	0.024 (2)	−0.0028 (17)	−0.0043 (18)	0.0001 (16)
C10	0.116 (6)	0.025 (3)	0.078 (5)	0.003 (3)	−0.056 (4)	0.009 (3)
C11	0.089 (5)	0.039 (3)	0.027 (3)	0.004 (2)	−0.029 (3)	−0.001 (2)
C12	0.040 (4)	0.065 (4)	0.053 (5)	0.017 (3)	−0.008 (3)	−0.015 (3)
O11	0.0229 (18)	0.0383 (17)	0.024 (2)	0.0050 (15)	−0.0012 (15)	0.0031 (16)
O12	0.062 (3)	0.036 (2)	0.034 (2)	−0.0029 (19)	−0.015 (2)	−0.0038 (17)
O13	0.0214 (17)	0.0368 (17)	0.023 (2)	−0.0041 (15)	−0.0035 (15)	0.0007 (15)
O14	0.069 (3)	0.044 (2)	0.033 (3)	0.008 (2)	−0.011 (2)	−0.0001 (19)

### Poly[[tetra-µ-aqua-diaquabis[3-(1,1,1-trimethylhydrazin-1-ium-2-yl)\ propanoate]disodium] dibromide tetrahydrate] (I) Geometric parameters (Å, º)

**Table:**

Na1—O1	2.367 (4)	N4—C12	1.530 (10)
Na1—O5	2.368 (3)	C1—C2	1.531 (6)
Na1—O6	2.369 (3)	C2—H2A	0.9900
Na1—O8^i^	2.517 (4)	C2—H2B	0.9900
Na1—O9	2.442 (4)	C2—C3	1.514 (6)
Na1—O10	2.361 (4)	C3—H3A	0.9900
Na2—O3	2.359 (4)	C3—H3B	0.9900
Na2—O5^ii^	2.543 (4)	C4—H4A	0.9800
Na2—O7	2.368 (3)	C4—H4B	0.9800
Na2—O8	2.364 (3)	C4—H4C	0.9800
Na2—O9	2.361 (3)	C5—H5C	0.9800
Na2—O10	2.449 (4)	C5—H5D	0.9800
O1—C1	1.241 (5)	C5—H5E	0.9800
O2—C1	1.281 (5)	C6—H6C	0.9800
O3—C7	1.249 (5)	C6—H6D	0.9800
O4—C7	1.282 (5)	C6—H6E	0.9800
O5—H5A	0.97 (6)	C7—C8	1.519 (6)
O5—H5B	0.82 (6)	C8—H8C	0.9900
O6—H6A	0.80 (3)	C8—H8D	0.9900
O6—H6B	0.82 (3)	C8—C9	1.506 (6)
O7—H7A	0.82 (3)	C9—H9C	0.9900
O7—H7B	0.80 (3)	C9—H9D	0.9900
O8—H8A	0.81 (3)	C10—H10C	0.9800
O8—H8B	0.81 (3)	C10—H10D	0.9800
O9—H9A	0.93 (6)	C10—H10E	0.9800
O9—H9B	0.79 (6)	C11—H11A	0.9800
O10—H10A	0.78 (6)	C11—H11B	0.9800
O10—H10B	0.90 (6)	C11—H11C	0.9800
N1—H1	0.83 (4)	C12—H12A	0.9800
N1—N2	1.471 (6)	C12—H12B	0.9800
N1—C3	1.468 (6)	C12—H12C	0.9800
N2—C4	1.499 (6)	O11—H11D	0.80 (3)
N2—C5	1.498 (8)	O11—H11E	0.80 (3)
N2—C6	1.489 (8)	O12—H12D	0.80 (3)
N3—H3	0.84 (4)	O12—H12E	0.80 (3)
N3—N4	1.466 (6)	O13—H13A	0.80 (3)
N3—C9	1.481 (6)	O13—H13B	0.80 (3)
N4—C10	1.463 (7)	O14—H14A	0.87 (5)
N4—C11	1.497 (8)	O14—H14B	0.87 (5)
			
O1—Na1—O5	109.25 (14)	N3—N4—C11	105.8 (4)
O1—Na1—O6	100.00 (12)	N3—N4—C12	105.9 (4)
O1—Na1—O8^i^	160.43 (13)	C10—N4—N3	114.7 (4)
O1—Na1—O9	83.01 (11)	C10—N4—C11	109.1 (5)
O5—Na1—O6	80.32 (12)	C10—N4—C12	111.6 (5)
O5—Na1—O8^i^	89.64 (11)	C11—N4—C12	109.5 (5)
O5—Na1—O9	167.30 (15)	O1—C1—O2	124.5 (4)
O6—Na1—O8^i^	87.89 (13)	O1—C1—C2	119.6 (4)
O6—Na1—O9	101.22 (13)	O2—C1—C2	115.9 (4)
O9—Na1—O8^i^	77.87 (13)	C1—C2—H2A	108.7
O10—Na1—O1	92.84 (12)	C1—C2—H2B	108.7
O10—Na1—O5	87.35 (12)	H2A—C2—H2B	107.6
O10—Na1—O6	164.52 (13)	C3—C2—C1	114.2 (4)
O10—Na1—O8^i^	82.69 (12)	C3—C2—H2A	108.7
O10—Na1—O9	88.81 (11)	C3—C2—H2B	108.7
O3—Na2—O5^ii^	160.55 (13)	N1—C3—C2	107.7 (3)
O3—Na2—O7	100.26 (12)	N1—C3—H3A	110.2
O3—Na2—O8	109.70 (14)	N1—C3—H3B	110.2
O3—Na2—O9	93.04 (13)	C2—C3—H3A	110.2
O3—Na2—O10	83.44 (11)	C2—C3—H3B	110.2
O5^ii^—Na2—H9A	71.3 (14)	H3A—C3—H3B	108.5
O7—Na2—O5^ii^	87.40 (13)	N2—C4—H4A	109.5
O7—Na2—H9A	143.9 (13)	N2—C4—H4B	109.5
O7—Na2—O10	100.79 (13)	N2—C4—H4C	109.5
O8—Na2—O5^ii^	89.11 (11)	H4A—C4—H4B	109.5
O8—Na2—O7	80.45 (11)	H4A—C4—H4C	109.5
O8—Na2—O10	166.49 (15)	H4B—C4—H4C	109.5
O9—Na2—O5^ii^	82.56 (12)	N2—C5—H5C	109.5
O9—Na2—O7	164.42 (13)	N2—C5—H5D	109.5
O9—Na2—O8	87.49 (12)	N2—C5—H5E	109.5
O9—Na2—O10	88.67 (11)	H5C—C5—H5D	109.5
O10—Na2—O5^ii^	77.55 (12)	H5C—C5—H5E	109.5
C1—O1—Na1	124.7 (2)	H5D—C5—H5E	109.5
C7—O3—Na2	124.5 (3)	N2—C6—H6C	109.5
Na1—O5—Na2^i^	90.26 (13)	N2—C6—H6D	109.5
Na1—O5—H5A	123 (3)	N2—C6—H6E	109.5
Na1—O5—H5B	132 (4)	H6C—C6—H6D	109.5
Na2^i^—O5—H5A	106 (4)	H6C—C6—H6E	109.5
Na2^i^—O5—H5B	93 (4)	H6D—C6—H6E	109.5
H5A—O5—H5B	102 (5)	O3—C7—O4	124.2 (4)
Na1—O6—H6A	124 (4)	O3—C7—C8	119.3 (4)
Na1—O6—H6B	120 (4)	O4—C7—C8	116.5 (4)
H6A—O6—H6B	108 (6)	C7—C8—H8C	108.4
Na2—O7—H7A	117 (4)	C7—C8—H8D	108.4
Na2—O7—H7B	122 (4)	H8C—C8—H8D	107.5
H7A—O7—H7B	105 (5)	C9—C8—C7	115.3 (4)
Na1^ii^—O8—H8A	103 (4)	C9—C8—H8C	108.4
Na1^ii^—O8—H8B	94 (5)	C9—C8—H8D	108.4
Na2—O8—Na1^ii^	91.00 (13)	N3—C9—C8	108.6 (4)
Na2—O8—H8A	126 (4)	N3—C9—H9C	110.0
Na2—O8—H8B	120 (4)	N3—C9—H9D	110.0
H8A—O8—H8B	111 (6)	C8—C9—H9C	110.0
Na1—O9—H9A	132 (4)	C8—C9—H9D	110.0
Na1—O9—H9B	107 (4)	H9C—C9—H9D	108.3
Na2—O9—Na1	91.34 (13)	N4—C10—H10C	109.5
Na2—O9—H9A	96 (4)	N4—C10—H10D	109.5
Na2—O9—H9B	123 (4)	N4—C10—H10E	109.5
H9A—O9—H9B	108 (5)	H10C—C10—H10D	109.5
Na1—O10—Na2	91.18 (13)	H10C—C10—H10E	109.5
Na1—O10—H10A	127 (4)	H10D—C10—H10E	109.5
Na1—O10—H10B	112 (4)	N4—C11—H11A	109.5
Na2—O10—H10A	102 (4)	N4—C11—H11B	109.5
Na2—O10—H10B	115 (4)	N4—C11—H11C	109.5
H10A—O10—H10B	108 (6)	H11A—C11—H11B	109.5
N2—N1—H1	104 (5)	H11A—C11—H11C	109.5
C3—N1—H1	106 (5)	H11B—C11—H11C	109.5
C3—N1—N2	113.3 (3)	N4—C12—H12A	109.5
N1—N2—C4	114.0 (4)	N4—C12—H12B	109.5
N1—N2—C5	106.6 (4)	N4—C12—H12C	109.5
N1—N2—C6	107.7 (4)	H12A—C12—H12B	109.5
C5—N2—C4	108.7 (4)	H12A—C12—H12C	109.5
C6—N2—C4	110.1 (4)	H12B—C12—H12C	109.5
C6—N2—C5	109.6 (4)	H11D—O11—H11E	107 (8)
N4—N3—H3	108 (4)	H12D—O12—H12E	111 (9)
N4—N3—C9	113.9 (3)	H13A—O13—H13B	102 (8)
C9—N3—H3	106 (5)	H14A—O14—H14B	113 (8)
			
Na1—O1—C1—O2	99.2 (4)	N4—N3—C9—C8	−167.1 (4)
Na1—O1—C1—C2	−78.2 (4)	C1—C2—C3—N1	−73.2 (5)
Na2—O3—C7—O4	−99.5 (4)	C3—N1—N2—C4	43.8 (6)
Na2—O3—C7—C8	78.8 (4)	C3—N1—N2—C5	163.8 (4)
O1—C1—C2—C3	−12.3 (5)	C3—N1—N2—C6	−78.6 (5)
O2—C1—C2—C3	170.1 (4)	C7—C8—C9—N3	73.4 (5)
O3—C7—C8—C9	10.5 (6)	C9—N3—N4—C10	−42.9 (7)
O4—C7—C8—C9	−171.1 (4)	C9—N3—N4—C11	−163.1 (5)
N2—N1—C3—C2	165.2 (4)	C9—N3—N4—C12	80.7 (5)

Symmetry codes: (i) *x*, *y*+1, *z*; (ii) *x*, *y*−1, *z*.

### Poly[[tetra-µ-aqua-diaquabis[3-(1,1,1-trimethylhydrazin-1-ium-2-yl)\ propanoate]disodium] dibromide tetrahydrate] (I) Hydrogen-bond geometry (Å, º)

**Table:**

*D*—H···*A*	*D*—H	H···*A*	*D*···*A*	*D*—H···*A*
O5—H5*A*···O1^i^	0.97 (6)	1.79 (6)	2.746 (4)	172 (6)
O5—H5*B*···O11^iii^	0.82 (6)	2.02 (6)	2.819 (5)	167 (6)
O6—H6*A*···O4^iv^	0.80 (3)	2.06 (3)	2.845 (5)	165 (6)
O6—H6*B*···O2^i^	0.82 (3)	1.91 (3)	2.732 (4)	175 (6)
O7—H7*A*···O2^v^	0.82 (3)	2.05 (3)	2.856 (5)	165 (6)
O7—H7*B*···O4^ii^	0.80 (3)	1.94 (3)	2.731 (4)	169 (6)
O8—H8*A*···O13^ii^	0.81 (3)	2.06 (3)	2.815 (5)	155 (5)
O8—H8*B*···O3^ii^	0.81 (3)	1.95 (3)	2.754 (4)	168 (7)
O9—H9*A*···O6^ii^	0.93 (6)	1.96 (6)	2.852 (4)	160 (5)
O9—H9*B*···O13	0.79 (6)	2.01 (6)	2.772 (5)	160 (6)
O10—H10*A*···O11^v^	0.78 (6)	2.00 (6)	2.771 (5)	167 (6)
O10—H10*B*···O7^i^	0.90 (6)	1.99 (6)	2.853 (4)	158 (5)
O11—H11*D*···O12	0.80 (3)	1.94 (3)	2.744 (6)	179 (7)
O11—H11*E*···O2	0.80 (3)	1.92 (3)	2.719 (5)	174 (9)
O13—H13*A*···O14	0.80 (3)	1.95 (3)	2.733 (6)	170 (6)
O13—H13*B*···O4^iv^	0.80 (3)	1.94 (3)	2.727 (5)	168 (9)
N1—H1···Br1^i^	0.83 (5)	2.57 (5)	3.379 (5)	167 (5)
N3—H3···Br2^v^	0.84 (5)	2.57 (5)	3.394 (5)	169 (5)
O12—H12*D*···Br1^i^	0.80 (5)	2.52 (6)	3.316 (4)	172 (6)
O12—H12*E*···Br1	0.80 (5)	2.49 (6)	3.289 (4)	177 (8)
O14—H14*A*···Br2^i^	0.87 (7)	2.47 (7)	3.323 (5)	168 (7)
O14—H14*B*···Br2	0.87 (6)	2.41 (6)	3.281 (5)	175 (6)

Symmetry codes: (i) *x*, *y*+1, *z*; (ii) *x*, *y*−1, *z*; (iii) *x*−1/2, −*y*+2, *z*; (iv) *x*+1/2, −*y*+2, *z*; (v) *x*−1/2, −*y*+1, *z*.

### Poly[[di-µ-aqua-diaquabis[µ-3-(1,1,1-trimethylhydrazin-1-ium-2-yl)\ propanoate]disodium] diiodide] (II) Crystal data

**Table:**

[Na_2_(C_6_H_14_N_2_O_2_)_2_(H_2_O)_4_]·I_2_	*F*(000) = 1312
*M**_r_* = 664.23	*D*_x_ = 1.779 Mg m^−^^3^
Monoclinic, *P*2_1_/*c*	Mo *K*α radiation, λ = 0.71073 Å
*a* = 19.7455 (11) Å	Cell parameters from 9932 reflections
*b* = 11.4530 (7) Å	θ = 3.1–27.9°
*c* = 10.9733 (7) Å	µ = 2.61 mm^−^^1^
β = 92.382 (2)°	*T* = 173 K
*V* = 2479.4 (3) Å^3^	Plate, colourless
*Z* = 4	0.3 × 0.2 × 0.07 mm

### Poly[[di-µ-aqua-diaquabis[µ-3-(1,1,1-trimethylhydrazin-1-ium-2-yl)\ propanoate]disodium] diiodide] (II) Data collection

**Table:**

Bruker PHOTON-100 CMOS diffractometer	5012 reflections with *I* > 2σ(*I*)
Radiation source: sealedtube	*R*_int_ = 0.048
φ and ω scans	θ_max_ = 27.1°, θ_min_ = 2.7°
Absorption correction: multi-scan (*TWINABS*; Krause *et al.*, 2015)	*h* = −25→25
*T*_min_ = 0.301, *T*_max_ = 0.431	*k* = 0→14
5475 measured reflections	*l* = 0→14
5475 independent reflections	

### Poly[[di-µ-aqua-diaquabis[µ-3-(1,1,1-trimethylhydrazin-1-ium-2-yl)\ propanoate]disodium] diiodide] (II) Refinement

**Table:**

Refinement on *F*^2^	0 restraints
Least-squares matrix: full	Hydrogen site location: mixed
*R*[*F*^2^ > 2σ(*F*^2^)] = 0.026	H atoms treated by a mixture of independent and constrained refinement
*wR*(*F*^2^) = 0.057	*w* = 1/[σ^2^(*F*_o_^2^) + (0.0177*P*)^2^ + 4.3911*P*] where *P* = (*F*_o_^2^ + 2*F*_c_^2^)/3
*S* = 1.17	(Δ/σ)_max_ = 0.001
5475 reflections	Δρ_max_ = 0.70 e Å^−^^3^
286 parameters	Δρ_min_ = −0.55 e Å^−^^3^

### Poly[[di-µ-aqua-diaquabis[µ-3-(1,1,1-trimethylhydrazin-1-ium-2-yl)\ propanoate]disodium] diiodide] (II) Special details

**Table:**

Geometry. All esds (except the esd in the dihedral angle between two l.s. planes) are estimated using the full covariance matrix. The cell esds are taken into account individually in the estimation of esds in distances, angles and torsion angles; correlations between esds in cell parameters are only used when they are defined by crystal symmetry. An approximate (isotropic) treatment of cell esds is used for estimating esds involving l.s. planes.
Refinement. Refined as a 2-component twin.

### Poly[[di-µ-aqua-diaquabis[µ-3-(1,1,1-trimethylhydrazin-1-ium-2-yl)\ propanoate]disodium] diiodide] (II) Fractional atomic coordinates and isotropic or equivalent isotropic displacement parameters (Å^2^)

**Table:**

	*x*	*y*	*z*	*U*_iso_*/*U*_eq_	
I1	0.25574 (2)	0.48014 (2)	0.74401 (2)	0.01967 (6)	
I2	0.02717 (2)	0.01593 (2)	0.75216 (2)	0.02095 (7)	
Na1	0.45889 (8)	0.30771 (15)	0.87401 (15)	0.0228 (4)	
Na2	0.54574 (8)	0.37173 (13)	0.56036 (14)	0.0177 (3)	
H5A	0.412 (3)	0.150 (5)	1.034 (5)	0.048 (18)*	
O1	0.38099 (12)	0.1816 (2)	0.7525 (3)	0.0205 (5)	
O2	0.29207 (14)	0.0789 (3)	0.8118 (2)	0.0219 (6)	
O3	0.55979 (13)	0.3377 (3)	0.7678 (3)	0.0252 (6)	
O4	0.57944 (14)	0.3107 (3)	0.9672 (2)	0.0193 (6)	
O5	0.43527 (15)	0.2101 (3)	1.0559 (3)	0.0212 (6)	
H5B	0.418 (3)	0.246 (5)	1.113 (5)	0.038 (15)*	
O6	0.42378 (16)	0.5067 (3)	0.8601 (3)	0.0273 (7)	
H6A	0.382266	0.512961	0.824831	0.041*	
H6B	0.428059	0.568362	0.908724	0.041*	
O7	0.63392 (14)	0.4980 (2)	0.5020 (3)	0.0225 (6)	
H7A	0.659951	0.465242	0.444491	0.034*	
H7B	0.662743	0.517998	0.565473	0.034*	
O8	0.49586 (16)	0.4327 (3)	0.3560 (3)	0.0225 (6)	
H8A	0.459 (3)	0.425 (5)	0.314 (6)	0.049 (17)*	
H8B	0.517 (3)	0.453 (5)	0.292 (7)	0.06 (2)*	
N1	0.16954 (18)	0.1758 (3)	0.6228 (3)	0.0167 (7)	
H1	0.137 (3)	0.143 (5)	0.651 (5)	0.044 (17)*	
N2	0.13426 (15)	0.2379 (3)	0.5206 (3)	0.0142 (7)	
N3	0.78736 (16)	0.2584 (3)	0.9016 (3)	0.0180 (7)	
H3	0.783 (3)	0.195 (5)	0.850 (5)	0.049 (17)*	
N4	0.83846 (16)	0.2233 (3)	0.9966 (3)	0.0183 (8)	
C1	0.32344 (17)	0.1332 (3)	0.7334 (4)	0.0155 (7)	
C2	0.29341 (19)	0.1380 (4)	0.6039 (4)	0.0202 (8)	
H2A	0.323168	0.092864	0.550794	0.024*	
H2B	0.294100	0.220190	0.576042	0.024*	
C3	0.22141 (19)	0.0918 (3)	0.5852 (4)	0.0186 (8)	
H3A	0.213016	0.072622	0.497921	0.022*	
H3B	0.216958	0.018875	0.632569	0.022*	
C4	0.0888 (2)	0.3230 (4)	0.5808 (4)	0.0227 (9)	
H4A	0.055992	0.280383	0.628483	0.034*	
H4B	0.064620	0.370023	0.518335	0.034*	
H4C	0.116052	0.374288	0.634871	0.034*	
C5	0.0924 (2)	0.1603 (4)	0.4372 (4)	0.0204 (9)	
H5C	0.122247	0.106328	0.395452	0.031*	
H5D	0.067248	0.208076	0.376722	0.031*	
H5E	0.060508	0.115656	0.484926	0.031*	
C6	0.1844 (2)	0.3039 (4)	0.4481 (4)	0.0197 (8)	
H6C	0.212631	0.352880	0.503036	0.029*	
H6D	0.160127	0.353351	0.387941	0.029*	
H6E	0.213223	0.248583	0.406033	0.029*	
C7	0.59886 (19)	0.3209 (3)	0.8594 (3)	0.0149 (8)	
C8	0.67425 (19)	0.3096 (3)	0.8349 (3)	0.0176 (8)	
H8C	0.680210	0.244686	0.776752	0.021*	
H8D	0.689360	0.382222	0.795342	0.021*	
C9	0.71953 (19)	0.2876 (3)	0.9479 (4)	0.0185 (8)	
H9A	0.722229	0.358018	1.000169	0.022*	
H9B	0.701879	0.221932	0.996013	0.022*	
C10	0.8943 (2)	0.1664 (4)	0.9296 (4)	0.0270 (10)	
H10A	0.876446	0.097529	0.885963	0.040*	
H10B	0.930579	0.142601	0.987910	0.040*	
H10C	0.912211	0.221802	0.871014	0.040*	
C11	0.8123 (2)	0.1393 (4)	1.0886 (4)	0.0309 (10)	
H11A	0.779170	0.178956	1.138310	0.046*	
H11B	0.850060	0.111068	1.141379	0.046*	
H11C	0.790487	0.073026	1.046252	0.046*	
C12	0.8657 (2)	0.3300 (4)	1.0583 (5)	0.0290 (10)	
H12A	0.883202	0.383425	0.997423	0.044*	
H12B	0.902343	0.308279	1.116894	0.044*	
H12C	0.829389	0.368612	1.101424	0.044*	

### Poly[[di-µ-aqua-diaquabis[µ-3-(1,1,1-trimethylhydrazin-1-ium-2-yl)\ propanoate]disodium] diiodide] (II) Atomic displacement parameters (Å^2^)

**Table:**

	*U*^11^	*U*^22^	*U*^33^	*U*^12^	*U*^13^	*U*^23^
I1	0.02077 (12)	0.02229 (12)	0.01602 (12)	−0.00185 (10)	0.00150 (12)	0.00076 (10)
I2	0.01730 (12)	0.02514 (12)	0.02035 (14)	−0.00498 (9)	−0.00011 (13)	0.00198 (10)
Na1	0.0199 (8)	0.0258 (9)	0.0226 (8)	−0.0008 (7)	−0.0001 (7)	0.0014 (7)
Na2	0.0173 (7)	0.0207 (8)	0.0151 (7)	−0.0017 (6)	0.0004 (6)	−0.0007 (6)
O1	0.0172 (13)	0.0235 (13)	0.0206 (13)	−0.0024 (10)	−0.0028 (11)	0.0040 (11)
O2	0.0199 (14)	0.0282 (16)	0.0178 (14)	−0.0012 (12)	0.0016 (11)	0.0040 (12)
O3	0.0190 (14)	0.0382 (16)	0.0181 (15)	0.0030 (11)	−0.0012 (12)	0.0042 (13)
O4	0.0182 (14)	0.0237 (15)	0.0160 (14)	−0.0010 (12)	0.0017 (11)	0.0015 (11)
O5	0.0166 (15)	0.0250 (16)	0.0223 (16)	−0.0002 (12)	0.0036 (12)	−0.0047 (13)
O6	0.0216 (16)	0.0324 (18)	0.0276 (16)	0.0020 (13)	−0.0013 (12)	−0.0066 (13)
O7	0.0194 (15)	0.0321 (17)	0.0162 (13)	−0.0044 (13)	0.0040 (11)	−0.0036 (12)
O8	0.0207 (17)	0.0298 (17)	0.0168 (15)	−0.0035 (13)	−0.0013 (13)	0.0015 (12)
N1	0.0169 (18)	0.0223 (18)	0.0107 (15)	−0.0012 (15)	−0.0017 (13)	0.0015 (13)
N2	0.0138 (14)	0.0175 (18)	0.0112 (17)	0.0014 (14)	−0.0011 (13)	−0.0009 (11)
N3	0.0145 (17)	0.0221 (18)	0.0173 (17)	0.0033 (14)	−0.0005 (13)	−0.0014 (13)
N4	0.0143 (15)	0.020 (2)	0.0202 (18)	0.0029 (12)	−0.0018 (14)	−0.0014 (14)
C1	0.0142 (17)	0.0135 (16)	0.0188 (18)	0.0056 (13)	0.0015 (15)	−0.0011 (14)
C2	0.014 (2)	0.027 (2)	0.019 (2)	−0.0001 (17)	0.0005 (16)	0.0008 (17)
C3	0.0186 (19)	0.018 (2)	0.0184 (19)	−0.0011 (16)	−0.0020 (16)	0.0012 (15)
C4	0.024 (2)	0.021 (2)	0.022 (2)	0.0074 (18)	0.0002 (18)	−0.0036 (17)
C5	0.021 (2)	0.024 (2)	0.0159 (19)	−0.0038 (18)	−0.0056 (16)	−0.0041 (16)
C6	0.018 (2)	0.022 (2)	0.019 (2)	−0.0033 (16)	−0.0001 (16)	0.0030 (16)
C7	0.0135 (19)	0.0137 (19)	0.0176 (18)	−0.0011 (15)	0.0006 (15)	0.0009 (14)
C8	0.019 (2)	0.0152 (19)	0.0182 (18)	0.0030 (15)	0.0011 (15)	0.0014 (14)
C9	0.0172 (19)	0.019 (2)	0.0194 (19)	0.0007 (15)	0.0032 (15)	0.0012 (15)
C10	0.015 (2)	0.034 (3)	0.032 (2)	0.0107 (19)	0.0003 (18)	−0.006 (2)
C11	0.027 (2)	0.042 (3)	0.023 (2)	0.006 (2)	−0.0025 (19)	0.013 (2)
C12	0.022 (2)	0.028 (3)	0.037 (3)	0.0025 (19)	−0.005 (2)	−0.011 (2)

### Poly[[di-µ-aqua-diaquabis[µ-3-(1,1,1-trimethylhydrazin-1-ium-2-yl)\ propanoate]disodium] diiodide] (II) Geometric parameters (Å, º)

**Table:**

Na1—Na2^i^	3.325 (2)	N3—N4	1.476 (4)
Na1—Na2	3.977 (2)	N3—C9	1.490 (5)
Na1—O1	2.462 (3)	N4—C10	1.499 (5)
Na1—O3	2.374 (3)	N4—C11	1.502 (5)
Na1—O4	2.552 (3)	N4—C12	1.487 (6)
Na1—O5	2.351 (3)	C1—C2	1.518 (5)
Na1—O6	2.385 (4)	C2—H2A	0.9900
Na1—O8^i^	2.857 (4)	C2—H2B	0.9900
Na1—C7	2.779 (4)	C2—C3	1.522 (5)
Na2—Na2^ii^	3.668 (3)	C3—H3A	0.9900
Na2—O3	2.315 (3)	C3—H3B	0.9900
Na2—O4^iii^	2.431 (3)	C4—H4A	0.9800
Na2—O5^iii^	2.373 (3)	C4—H4B	0.9800
Na2—O7	2.372 (3)	C4—H4C	0.9800
Na2—O8^ii^	2.569 (4)	C5—H5C	0.9800
Na2—O8	2.510 (3)	C5—H5D	0.9800
O1—C1	1.274 (4)	C5—H5E	0.9800
O2—C1	1.247 (4)	C6—H6C	0.9800
O3—C7	1.256 (5)	C6—H6D	0.9800
O4—C7	1.264 (5)	C6—H6E	0.9800
O5—H5A	0.86 (6)	C7—C8	1.529 (5)
O5—H5B	0.84 (6)	C8—H8C	0.9900
O6—H6A	0.8946	C8—H8D	0.9900
O6—H6B	0.8873	C8—C9	1.520 (5)
O7—H7A	0.9110	C9—H9A	0.9900
O7—H7B	0.9103	C9—H9B	0.9900
O8—H8A	0.85 (6)	C10—H10A	0.9800
O8—H8B	0.86 (7)	C10—H10B	0.9800
N1—H1	0.83 (6)	C10—H10C	0.9800
N1—N2	1.478 (4)	C11—H11A	0.9800
N1—C3	1.476 (5)	C11—H11B	0.9800
N2—C4	1.496 (5)	C11—H11C	0.9800
N2—C5	1.499 (5)	C12—H12A	0.9800
N2—C6	1.500 (5)	C12—H12B	0.9800
N3—H3	0.92 (6)	C12—H12C	0.9800
			
O1—Na1—O4	140.94 (12)	N3—N4—C10	105.5 (3)
O1—Na1—O8^i^	63.43 (9)	N3—N4—C11	113.9 (3)
O1—Na1—C7	126.95 (12)	N3—N4—C12	108.8 (3)
O3—Na1—O1	109.70 (11)	C10—N4—C11	109.4 (3)
O3—Na1—O4	53.63 (9)	C12—N4—C10	108.8 (3)
O3—Na1—O6	94.48 (12)	C12—N4—C11	110.3 (4)
O3—Na1—O8^i^	83.30 (11)	O1—C1—C2	116.7 (3)
O3—Na1—C7	26.77 (10)	O2—C1—O1	124.6 (4)
O4—Na1—O8^i^	78.70 (10)	O2—C1—C2	118.6 (3)
O4—Na1—C7	27.00 (10)	C1—C2—H2A	108.3
O5—Na1—O1	92.25 (11)	C1—C2—H2B	108.3
O5—Na1—O3	133.27 (12)	C1—C2—C3	116.1 (3)
O5—Na1—O4	83.16 (11)	H2A—C2—H2B	107.4
O5—Na1—O6	116.18 (13)	C3—C2—H2A	108.3
O5—Na1—O8^i^	70.18 (11)	C3—C2—H2B	108.3
O5—Na1—C7	107.91 (12)	N1—C3—C2	113.0 (3)
O6—Na1—O1	110.65 (11)	N1—C3—H3A	109.0
O6—Na1—O4	106.06 (12)	N1—C3—H3B	109.0
O6—Na1—O8^i^	172.17 (12)	C2—C3—H3A	109.0
O6—Na1—C7	103.39 (12)	C2—C3—H3B	109.0
C7—Na1—O8^i^	77.92 (11)	H3A—C3—H3B	107.8
O3—Na2—O4^iii^	104.18 (11)	N2—C4—H4A	109.5
O3—Na2—O5^iii^	91.51 (11)	N2—C4—H4B	109.5
O3—Na2—O7	108.00 (12)	N2—C4—H4C	109.5
O3—Na2—O8	162.13 (12)	H4A—C4—H4B	109.5
O3—Na2—O8^ii^	79.78 (11)	H4A—C4—H4C	109.5
O4^iii^—Na2—O8^ii^	175.55 (12)	H4B—C4—H4C	109.5
O4^iii^—Na2—O8	88.14 (11)	N2—C5—H5C	109.5
O5^iii^—Na2—O4^iii^	85.36 (11)	N2—C5—H5D	109.5
O5^iii^—Na2—O8^ii^	92.56 (12)	N2—C5—H5E	109.5
O5^iii^—Na2—O8	76.41 (11)	H5C—C5—H5D	109.5
O7—Na2—O4^iii^	101.17 (11)	H5C—C5—H5E	109.5
O7—Na2—O5^iii^	156.89 (13)	H5D—C5—H5E	109.5
O7—Na2—O8	81.63 (11)	N2—C6—H6C	109.5
O7—Na2—O8^ii^	79.28 (11)	N2—C6—H6D	109.5
O8—Na2—O8^ii^	87.55 (11)	N2—C6—H6E	109.5
C1—O1—Na1	151.3 (3)	H6C—C6—H6D	109.5
C7—O3—Na1	94.9 (2)	H6C—C6—H6E	109.5
C7—O3—Na2	149.0 (2)	H6D—C6—H6E	109.5
Na2^i^—O4—Na1	83.67 (10)	O3—C7—Na1	58.34 (19)
C7—O4—Na1	86.6 (2)	O3—C7—O4	124.2 (3)
C7—O4—Na2^i^	124.9 (3)	O3—C7—C8	116.2 (3)
Na1—O5—Na2^i^	89.48 (11)	O4—C7—Na1	66.4 (2)
Na1—O5—H5A	105 (4)	O4—C7—C8	119.5 (3)
Na1—O5—H5B	120 (4)	C8—C7—Na1	169.5 (3)
Na2^i^—O5—H5A	99 (4)	C7—C8—H8C	108.6
Na2^i^—O5—H5B	126 (4)	C7—C8—H8D	108.6
H5A—O5—H5B	112 (5)	H8C—C8—H8D	107.6
Na1—O6—H6A	111.3	C9—C8—C7	114.5 (3)
Na1—O6—H6B	134.6	C9—C8—H8C	108.6
H6A—O6—H6B	105.0	C9—C8—H8D	108.6
Na2—O7—H7A	112.2	N3—C9—C8	105.4 (3)
Na2—O7—H7B	112.9	N3—C9—H9A	110.7
H7A—O7—H7B	106.3	N3—C9—H9B	110.7
Na1^iii^—O8—H8A	74 (4)	C8—C9—H9A	110.7
Na1^iii^—O8—H8B	117 (4)	C8—C9—H9B	110.7
Na2—O8—Na1^iii^	76.24 (10)	H9A—C9—H9B	108.8
Na2^ii^—O8—Na1^iii^	136.82 (13)	N4—C10—H10A	109.5
Na2—O8—Na2^ii^	92.45 (11)	N4—C10—H10B	109.5
Na2—O8—H8A	139 (4)	N4—C10—H10C	109.5
Na2^ii^—O8—H8A	90 (4)	H10A—C10—H10B	109.5
Na2—O8—H8B	129 (4)	H10A—C10—H10C	109.5
Na2^ii^—O8—H8B	103 (4)	H10B—C10—H10C	109.5
H8A—O8—H8B	90 (5)	N4—C11—H11A	109.5
N2—N1—H1	99 (4)	N4—C11—H11B	109.5
C3—N1—H1	112 (4)	N4—C11—H11C	109.5
C3—N1—N2	114.3 (3)	H11A—C11—H11B	109.5
N1—N2—C4	104.6 (3)	H11A—C11—H11C	109.5
N1—N2—C5	114.1 (3)	H11B—C11—H11C	109.5
N1—N2—C6	110.1 (3)	N4—C12—H12A	109.5
C4—N2—C5	109.3 (3)	N4—C12—H12B	109.5
C4—N2—C6	109.1 (3)	N4—C12—H12C	109.5
C5—N2—C6	109.5 (3)	H12A—C12—H12B	109.5
N4—N3—H3	105 (4)	H12A—C12—H12C	109.5
N4—N3—C9	114.7 (3)	H12B—C12—H12C	109.5
C9—N3—H3	109 (4)		
			
Na1—O1—C1—O2	−48.2 (7)	O2—C1—C2—C3	9.4 (5)
Na1—O1—C1—C2	134.9 (4)	O3—C7—C8—C9	−179.1 (3)
Na1—O3—C7—O4	−8.8 (4)	O4—C7—C8—C9	−0.5 (5)
Na1—O3—C7—C8	169.7 (3)	N2—N1—C3—C2	102.5 (4)
Na1—O4—C7—O3	8.2 (4)	N4—N3—C9—C8	−174.9 (3)
Na1—O4—C7—C8	−170.3 (3)	C1—C2—C3—N1	78.7 (4)
Na1—C7—C8—C9	−122.6 (14)	C3—N1—N2—C4	−175.9 (3)
Na2—O3—C7—Na1	−175.0 (6)	C3—N1—N2—C5	64.8 (4)
Na2—O3—C7—O4	176.2 (3)	C3—N1—N2—C6	−58.8 (4)
Na2—O3—C7—C8	−5.3 (7)	C7—C8—C9—N3	170.1 (3)
Na2^i^—O4—C7—Na1	79.9 (2)	C9—N3—N4—C10	164.3 (3)
Na2^i^—O4—C7—O3	88.0 (5)	C9—N3—N4—C11	44.3 (4)
Na2^i^—O4—C7—C8	−90.4 (4)	C9—N3—N4—C12	−79.2 (4)
O1—C1—C2—C3	−173.5 (3)		

Symmetry codes: (i) *x*, −*y*+1/2, *z*+1/2; (ii) −*x*+1, −*y*+1, −*z*+1; (iii) *x*, −*y*+1/2, *z*−1/2.

### Poly[[di-µ-aqua-diaquabis[µ-3-(1,1,1-trimethylhydrazin-1-ium-2-yl)\ propanoate]disodium] diiodide] (II) Hydrogen-bond geometry (Å, º)

**Table:**

*D*—H···*A*	*D*—H	H···*A*	*D*···*A*	*D*—H···*A*
O5—H5*B*···O1^i^	0.85 (6)	1.89 (6)	2.741 (4)	175 (6)
O7—H7*B*···O2^iv^	0.91	1.73	2.629 (4)	169
O8—H8*A*···O1^iii^	0.85 (6)	2.05 (6)	2.815 (4)	149 (6)
N1—H1···I2	0.82 (6)	2.87 (6)	3.688 (4)	177 (5)
N3—H3···I1^v^	0.92 (6)	2.76 (6)	3.650 (3)	161 (5)
O5—H5*A*···O7^v^	0.86 (6)	2.00 (6)	2.846 (4)	172 (4)
O6—H6*A*···I1	0.89	2.64	3.518 (3)	166
O6—H6*B*···O4^vi^	0.89	1.95	2.825 (4)	168
O7—H7*A*···I1^ii^	0.91	2.78	3.548 (3)	143
O8—H8*B*···O6^ii^	0.86 (7)	2.13 (7)	2.989 (5)	175 (5)
C3—H3*A*···I1^iii^	0.99	3.01	3.920 (4)	154
C11—H11*B*···I2^vii^	0.98	3.02	3.975 (4)	165
C12—H12*C*···I1^vi^	0.98	2.99	3.952 (5)	167

Symmetry codes: (i) *x*, −*y*+1/2, *z*+1/2; (ii) −*x*+1, −*y*+1, −*z*+1; (iii) *x*, −*y*+1/2, *z*−1/2; (iv) −*x*+1, *y*+1/2, −*z*+3/2; (v) −*x*+1, *y*−1/2, −*z*+3/2; (vi) −*x*+1, −*y*+1, −*z*+2; (vii) −*x*+1, −*y*, −*z*+2.
